# Sex-specific differences in the associations between adiposity indices and incident hyperuricemia among middle-aged and older adults: a nationwide longitudinal study

**DOI:** 10.3389/fendo.2024.1336471

**Published:** 2024-02-09

**Authors:** Zhiyi Liu, Qinwu Zhou, Yuqiong Tang, Jiyong Li, Qiutong Chen, Hongguang Yang, Shuhan Zhou

**Affiliations:** ^1^College of Traditional Chinese Medicine, Hubei University of Chinese Medicine, Wuhan, China; ^2^Department of Dermatology, Tongji Hospital, Tongji Medical College, Huazhong University of Science and Technology, Wuhan, China; ^3^The First Clinical Medical School, Hubei University of Chinese Medicine, Wuhan, China; ^4^Huangpi Hospital of Traditional Chinese Medicine, Wuhan, China; ^5^College of Language Intelligence, Sichuan International Studies University, Chongqing, China; ^6^Department of Clinical Nutrition, Union Shenzhen Hospital of Huazhong University of Science and Technology, Shenzhen, Guangdong, China; ^7^Hubei Shizhen Laboratory, Hubei University of Chinese Medicine, Wuhan, China

**Keywords:** obesity, adiposity indices, hyperuricemia, longitudinal study, CHARLS

## Abstract

**Objective:**

Although obesity is a known risk for hyperuricemia (HUA), the associations between adiposity indices and incident HUA and whether sex-specific differences exist is still unknown. We aimed to investigate the associations between adiposity indices and incident HUA in a longitudinal study.

**Methods:**

Data from the China Health and Retirement Longitudinal Study (CHARLS) in 2011–2012 and 2015–2016 were used to conduct a cohort study. Participants aged ≥45 years without HUA at baseline were included in this study. Adiposity indices, including body mass index (BMI), waist circumference (WC), waist-to-height ratio body roundness index (BRI), conicity index (CI), lipid accumulation product (LAP) index, waist-to-height ratio (WHtR), visceral adiposity index (VAI), and Chinese visceral adiposity index (CVAI), were calculated. Logistic analysis was used to analyze the association between adiposity indices and incident HUA risk stratified by gender. Receiver operating characteristic curve analysis was performed to evaluate the power of predictions for incident HUA.

**Results:**

Of 5,873 participants aged 59.0 ± 8.7 years enrolled in this study, 578 (9.8%) participants developed HUA during the 4-year follow-up period. After adjusting for confounding variables, LAP, VAI, and CVAI showed significant association with incident HUA. BMI, WC, WHtR, BRI, and CI were significantly associated with incident HUA in women but not in men. LAP had the highest area under the curve (AUC) (0.612) followed by CVAI (0.596) in men, while CVAI had the highest AUC (0.707) followed by LAP (0.691) in women. All indices showed better predictive ability in women than in men.

**Conclusion:**

Our findings indicated that adiposity indices were effective predictors of incident HUA and showed better predictive power in women than men. In clinical practice, adiposity indices could be used to assess and prevent incident HUA among Chinese middle-aged and older adults.

## Introduction

Uric acid is the end enzymatic product of purine metabolism (1). Hyperuricemia (HUA) is the elevation of serum uric acid (SUA) concentrations, usually resulting from excessive production or reduced urinary excretion of uric acid (1, 2). In recent years, HUA has become a critical public health issue worldwide. It was reported that 14.0% of Chinese adults had HUA during 2018–2019 according to a nationwide study (3), and approximately 20% of American adults had HUA according to the National Health and Nutrition Examination Survey (4). HUA is a known risk factor for gout, diabetes, chronic kidney disease, hypertension, cardiovascular diseases, and all-cause mortality (5–7). Given the increasing number of people with HUA, it is important to understand the modifiable risk factors and well-predictive indicators for HUA in clinical practice.

Although the impact of uric acid on metabolic syndrome has been thoroughly studied (8), only a few studies to date have linked obesity to serum uric acid and incident HUA (9–12). Accumulating evidence demonstrates obesity, especially visceral obesity, is positively associated with SUA levels and HUA (9). Obesity is highly related to metabolic disorders, which is a major risk of HUA. Ryu et al. reported that obesity was a primary determinant risk factor of HUA in middle-aged South Korean men (13). Moreover, obese individuals, especially those with abdominal obesity, displayed a significantly increased risk of HUA (9–11). In recent years, many adiposity indices have been developed to evaluate obesity status, body fat distribution, and visceral adiposity proportions (14). The associations between these adiposity indices and HUA have been explored in cross-sectional studies (11, 15, 16). However, these studies yielded inconsistent results. Furthermore, some traditional adiposity indices without lipid parameters such as body mass index (BMI), waist circumference (WC), waist-to-height ratio (WHtR), and body roundness index (BRI) reported inconsistent associations in different studies, especially in different genders (11, 15, 16). However, several novel adiposity indices containing lipid parameters such as lipid accumulation product (LAP) index and visceral adiposity index (VAI), which have shown stronger correlations with metabolic abnormality, consistently show a positive association with HUA (9, 11). In addition, previous studies reported the effect of cumulative burden of abnormal VAI, and its components were more pronounced in women (17), and several adiposity indices showed higher predictive power for HUA in women (15, 16), which suggested that there might be gender differences in relationships between adiposity indices and HUA.

Although several cross-sectional studies have explored the associations between adiposity indices and HUA, the results were still mixed, and longitudinal evidence was lacking (9, 11, 12, 18). Therefore, we aimed to investigate the relationship between adiposity indices and incident HUA in a cohort of Chinese middle-aged and older adults, which may provide the basis for individuals to prevent the incidence of HUA.

## Methods

### Study population

Data used in this study were from the China Health and Retirement Longitudinal Study (CHARLS), an ongoing national representative survey conducted in China. Details of the study could be found elsewhere (19). Briefly, this survey recruited participants from 450 urban communities and rural areas in 28 provinces of China from 2011 to 2012 (Wave 1). They were followed up every 2 years subsequently. In the present study, data collected at Wave 1 and Wave 3 (2015 to 2016) were used because data on laboratory blood samples were only available in the two waves.

**Figure 1:**
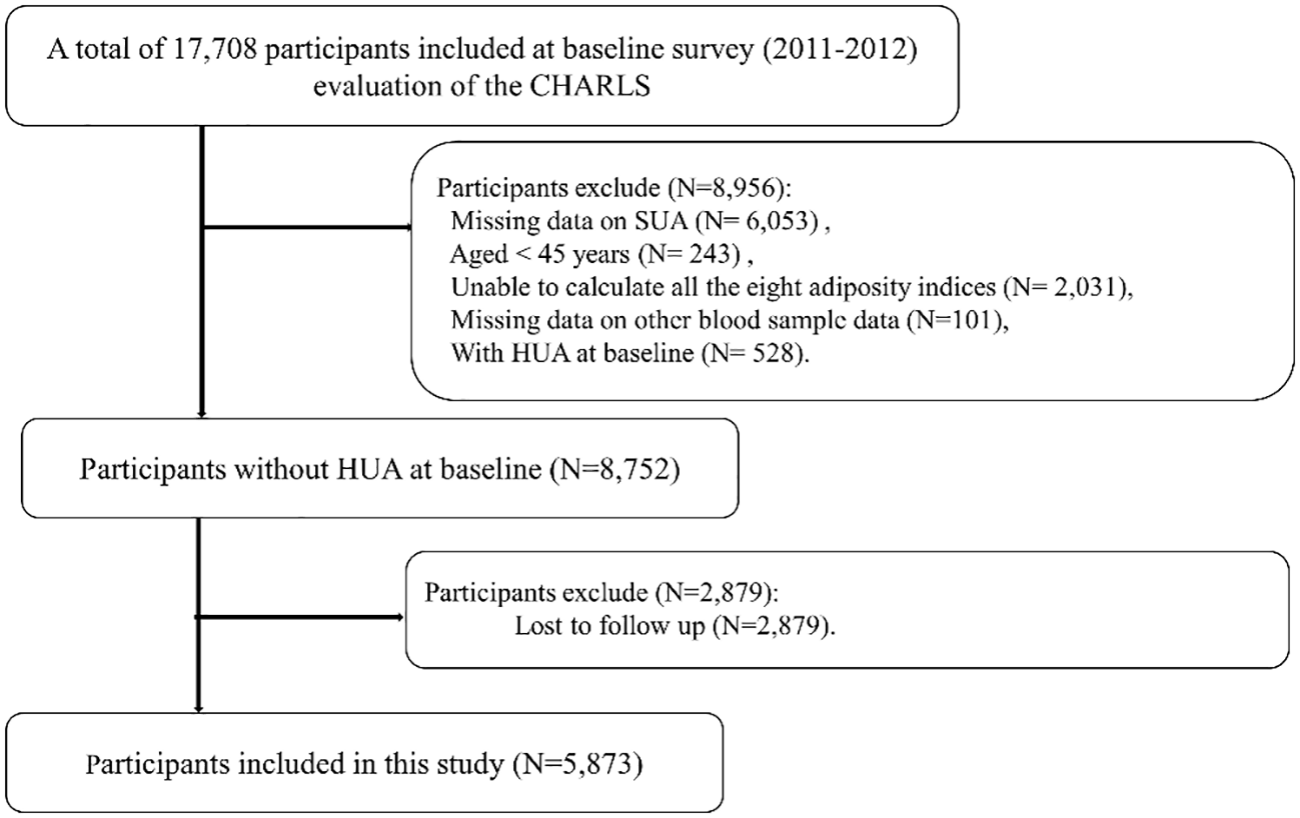
Flowchart of study population.

Of 17,708 participants, we excluded 6,053 participants with missing data on SUA, 243 participants aged <45 years, 2,031 participants in whom all the eight adiposity indices were unable to be calculated, 101 participants with missing data on other blood tests such as blood urea nitrogen (BUN) and plasma creatinine, and 528 participants with HUA at baseline in Wave 1. We further excluded 2,879 participants lost to follow-up in Wave 3. Finally, we included a total of 5,873 participants in the present study. Details regarding the study population selection are provided in **Figure 1**.

### Anthropometric and laboratory measurements

Face-to-face interviews were performed to collect data on demographic information. Participants reported their age, gender, educational levels (primary school or below, middle school, and high school or above), places of residence (rural and urban), marital status, and history of smoking and alcohol drinking. Their height, weight, and WC were measured by trained investigators using standard methods.

Fasting blood samples were collected to measure the blood biochemical indices, including SUA, fasting plasma glucose (FPG), glycosylated hemoglobin (HbA1c), total cholesterol (TC), triglyceride (TG), high-density lipoprotein-cholesterol (HDL), low-density lipoprotein-cholesterol (LDL), C-reactive protein (CRP), blood urea nitrogen, and serum creatinine. SUA was measured using the UA plus method. FPG, TG, TC, HDL, and LDL were measured using an enzymatic colorimetric test method. The HbA1c assay was performed using the boronate-affinity high-performance liquid chromatography (HPLC) method. BUN was measured using the enzymatic ultraviolet method with urease. CRP was measured using an immunoturbidimetric assay. Serum creatinine was measured by rate-blanking and compensated Jaffe creatinine method.

### Definitions of HUA and adiposity indices

According to a previous study, HUA was defined as SUA ≥7.0 mg/dL in men and ≥6.0 mg/dL in women (20, 21).

Adiposity indices were calculated using formulas in **Table 1** and were then categorized into quantiles (14, 22, 23).

**Table 1:** Formulas of adiposity indices.

Adiposity index	Formula
BMI	weight_(kg)_/height^2^_(m_^2^_)_
WHtR	WC_(m)_/height_(m)_
CI	(WC_(m)_) ×0.109^−1^ × (weight_(kg)_/height_(m)_)^−1/2^
BRI	364.2 – 365.5 × [1 − [(WC/2π)/(0.5 × height)]^2^]^1/2^
LAP	TG_(mmol/L)_ × (WC_[cm]_ − 65) (men)
	TG_(mmol/L)_ × (WC_[cm]_ − 58) (women)
VAI	[(WC_(cm)_/[39.68 + (1.88 × BMI)]] × (TG_(mmol/L)_/1.03) × (1.31/HDL_(mmol/L)_) (men)
	[(WC_(cm)_/[36.58 + (1.889 × BMI)]] × (TG_(mmol/L)_/0.81) × (1.52/HDL_(mmol/L)_) (women)
CVAI	−267.93 + 0.68 × age + 0.03 × BMI + 4 × WC + 22 × logTG _(mmol/L)_ − 16.32 × HDL _(mmol/L)_ (men)
	−187.32 + 1.71 × age + 4.23 × BMI + 1.12 × WC + 39.76 × logTG _(mmol/L)_ − 11.66 × HDL _(mmol/L)_ (women)

BMI, body mass index; WC, waist circumference; WHtR, waist-to-height ratio; BRI, body roundness index; CI, conicity index; LAP, lipid accumulation product index; VAI, visceral adiposity index; CVAI, Chinese visceral adiposity index.

Hypertension was defined as self-reported hypertension, systolic blood pressure (SBP) ≥140 mmHg, and/or diastolic blood pressure (DBP) ≥90 mmHg.

### Statistical analysis

Continuous data are presented as means ± SD, and categorical data are presented as frequency (percentage). The differences between continuous variables and categorical variables were compared using t-test and chi-squared test, respectively. Logistic regression analyses were performed to examine the association between adiposity indices and incident HUA using three models. Model 1 was unadjusted. Model 2 was adjusted for age, educational levels, places of residence, drinking status, smoking status, marital status, and SBP. Model 3 was further adjusted for BMI, WC, BRI, WHtR, and conicity index (CI) and for LAP, VAI, and Chinese visceral adiposity index (CVAI), without TG. In addition, a sensitivity analysis was performed under the definition that HUA was defined as SUA ≥ 7.0 mg/dL in men and women.

The area under the curve (AUC) was calculated by receiver operating characteristic (ROC) curve analysis to compare the predictive ability of different adiposity indices for incident HUA. The cutoff value of adiposity indices calculated by ROC curve analysis was determined using the maximized Youden index value (24). Finally, chi-squared tests were used to determine differences in the incidence of HUA based on the cutoff values obtained by ROC analysis, and then Cramer’s V was applied to determine the interpreted effect size (23, 25). All data analyses were performed using SPSS version 24.0 (IBM Corp., Armonk, NY, USA) and R software (version 4.1.0, http://www.r-project.org). A two-sided *p*-value < 0.05 was considered statistically significant.

## Results

Of 5,873 participants (mean age, 59.0 ± 8.7 years) without HUA at baseline enrolled in this study, 2,669 (45.4%) were men. During the 4-year follow-up period, 578 (9.8%) participants developed HUA, with 296 (11.1%) in male participants and 282 (8.8%) in female participants.

### Baseline characteristics

The baseline characteristics of participants are summarized in **Table 2**. Compared to those without incident HUA, male participants with incident HUA had a higher prevalence of hypertension; higher SBP, DBP, SUA, serum creatinine, TC, and TG; and lower HDL. Female participants with incident HUA had higher age; higher prevalence of hypertension; higher SBP, DBP, SUA, CRP, serum creatinine, TC, TG, FPG, HbA1c, and BUN; and lower HDL. Regarding the adiposity indices, participants with incident HUA had higher levels of all eight adiposity indices than those without incident HUA in both male and female participants.

**Table 2:** Baseline characteristics of the study participants were classified by the presence of different genders and incident HUA.

	Male (N = 2,669)			Female (N = 3,204)		
	Not incident HUA (N = 2,373)	Incident HUA (N = 296)	*p*-Value	Not incident HUA (N = 2,922)	Incident HUA (N = 282)	*p*-Value
**Age, year**	60.0 ± 8.7	60.5 ± 9.0	0.341	57.9 ± 8.6	60.3 ± 9.4	<0.001
**Smoking, n (%)**	1,801 (75.9)	217 (73.3)	0.329	205 (7.0)	27 (9.6)	0.113
**Drinking, n (%)**	1,332 (56.1)	176 (59.5)	0.276	342 (11.7)	36 (12.8)	0.598
**Marriage status, n (%)**			0.410			0.064
Married	2,154 (90.8)	273 (92.2)		2,558 (87.5)	236 (83.7)	
Not married	219 (9.2)	23 (7.8)		364 (12.5)	46 (16.3)	
**Places of residence, n (%)**			0.143			0.183
Rural	2,032 (85.6)	244 (82.4)		2,433 (83.3)	226 (80.1)	
Urban	341 (14.4)	52 (17.6)		489 (16.7)	56 (19.9)	
**Educational level, n (%)**			0.526			0.988
Primary school and lower	1,449 (61.0)	190 (64.1)		2,321 (79.4)	225 (79.8)	
Middle school	630 (26.6)	75 (25.4)		424 (14.5)	40 (14.2)	
High school and above	294 (12.4)	31 (10.5)		177 (6.1)	17 (6.0)	
Hypertension, n (%)	880 (37.1)	156 (52.7)	<0.001	1,150 (39.4)	162 (57.4)	<0.001
**SBP**	129.0 ± 19.7	134.7 ± 22.5	<0.001	129.4 ± 22.5	136.0 ± 22.1	<0.001
**DBP**	75.6 ± 12.2	78.2 ± 12.9	<0.001	75.1 ± 11.8	78.4 ± 12.3	<0.001
**HDL (mmol/L)**	1.32 ± 0.42	1.25 ± 0.41	<0.001	1.35 ± 0.37	1.20 ± 0.36	<0.001
**LDL (mmol/L)**	2.9 ± 0.9	3.0 ± 0.9	0.125	3.1 ± 0.9	3.1 ± 1.1	0.548
**TG (mmol/L)**	1.4 ± 1.0	1.7 ± 1.3	<0.001	1.5 ± 1.0	2.0 ± 1.2	<0.001
**TC (mmol/L)**	4.8 ± 0.9	5.0 ± 0.9	<0.001	5.1 ± 1.0	5.2 ± 1.1	0.017
**FPG (mmol/L)**	6.1 ± 2.0	6.2 ± 2.0	0.556	6.1 ± 1.9	6.3 ± 2.1	0.033
**HbA1c, %**	5.2 ± 0.7	5.3 ± 0.8	0.672	5.3 ± 0.8	5.4 ± 1.0	0.007
**Creatinine (mg/dL)**	0.8 ± 0.2	0.9 ± 0.2	<0.001	0.7 ± 0.1	0.7 ± 0.1	<0.001
**CRP**	2.7 ± 8.1	2.7 ± 5.5	0.911	2.2 ± 5.8	3.2 ± 7.5	0.007
**BUN (mg/dL)**	16.4 ± 4.4	16.4 ± 4.0	0.798	14.8 ± 4.2	15.2 ± 3.9	0.014
**SUA (mg/dL)**	4.6 ± 1.0	5.7 ± 0.9	<0.001	3.8 ± 0.8	4.8 ± 0.7	<0.001
**BMI**	23.1 ± 11.2	23.8 ± 3.3	0.277	23.9 ± 4.0	25.8 ± 4.0	<0.001
**WC**	84.6 ± 9.4	87.4 ± 9.9	<0.001	85.3 ± 10.0	91.1 ± 9.6	<0.001
**CI**	1.27 ± 0.08	1.28 ± 0.08	<0.001	1.30 ± 0.09	1.33 ± 0.09	<0.001
**WHtR**	0.52 ± 0.07	0.53 ± 0.06	<0.001	0.56 ± 0.07	0.60 ± 0.06	<0.001
**BRI**	3.8 ± 1.8	4.1 ± 1.2	<0.001	4.6 ± 1.4	5.4 ± 1.3	<0.001
**LAP**	29.3 ± 35.2	40.3 ± 41.8	<0.001	42.9 ± 37.3	66.3 ± 47.2	<0.001
**VAI**	1.6 ± 2.3	2.3 ± 3.6	<0.001	2.7 ± 3.1	4.0 ± 4.4	<0.001
**CVAI**	91.6 ± 42.8	105.9 ± 44.9	<0.001	97.1 ± 34.3	122.2 ± 33.1	<0.001

HUA, hyperuricemia; SBP, systolic blood pressure; DBP, diastolic blood pressure; TG, triglycerides; HDL, high-density lipoprotein; TC, total cholesterol; LDL, low-density lipoprotein; FPG, fasting plasma glucose; HbA1c, glycosylated hemoglobin; CRP, C-reactive protein; BUN, blood urea nitrogen; SUA, serum uric acid; BMI, body mass index; WC, waist circumference; WHtR, waist-to-height ratio; BRI, body roundness index; CI, conicity index; LAP, lipid accumulation product index; VAI, visceral adiposity index; CVAI, Chinese visceral adiposity index.

### Associations between adiposity indices with incident HUA stratified by gender

The associations between adiposity indices with incident HUA in men are shown in **Table 3**. After adjusting for potential covariates, LAP, VAI, and CVAI were significantly associated with incident HUA. Compared with the first quartile, the hazard ratio (HR) of incident HUA in the highest quartile of the LAP was 1.821 (95%CIs, 1.18 to 2.852); the HR in the highest quartile of the VAI was 1.735 (95%CIs, 1.162 to 2.617); the HR in the highest quartile of the CVAI was 1.547 (95%CIs, 1.023 to 2.360).

**Table 3:** Association between adiposity indices with incident hyperuricemia multivariate logistic regression analysis in male participants.

		Model 1		Model 2		Model 3	
		HR (95%CIs)	*p*-Value	HR (95%CIs)	*p*-Value	HR (95%CIs)	*p*-Value
BMI	Q1 (<20.525)	1 (Ref)		1 (Ref)		1 (Ref)	
	Q2 (20.525–22.540)	1.501 (1.013, 2.240)	0.044	1.419 (0.959, 2.115)	0.082	1.378 (0.902, 2.117)	0.140
	Q3 (22.540–24.972)	2.029 (1.390, 2.994)	<0.001	1.843 (1.261, 2.721)	0.002	1.481 (0.979, 2.258)	0.065
	Q4 (>24.972)	2.487 (1.707, 3.672)	<0.001	2.099 (1.429, 3.119)	<0.001	1.421 (0.924, 2.204)	0.113
WC	Q1 (<78.00)	1 (Ref)		1 (Ref)		1 (Ref)	
	Q2 (78.00–84.00)	1.184 (0.804, 1.750)	0.393	1.130 (0.767, 1.668)	0.537	1.267 (0.835, 1.929)	0.267
	Q3 (84.00–91.20)	1.513 (1.046, 2.205)	0.029	1.376 (0.950, 2.008)	0.094	1.122 (0.747, 1.696)	0.581
	Q4 (>91.20)	2.123 (1.484, 3.067)	<0.001	1.818 (1.263, 2.641)	0.001	1.326 (0.877, 2.018)	0.184
WHtR	Q1 (<0.477)	1 (Ref)		1 (Ref)		1 (Ref)	
	Q2 (0.477–0.513)	1.293 (0.869, 1.933)	0.207	1.214 (0.817, 1.815)	0.339	1.140 (0.744, 1.753)	0.548
	Q3 (0.513–0.557)	1.845 (1.269, 2.709)	0.002	1.670 (1.147, 2.454)	0.008	1.295 (0.861, 1.962)	0.217
	Q4 (>0.557)	2.468 (1.718, 3.593)	<0.001	2.109 (1.457, 3.089)	<0.001	1.449 (0.959, 2.207)	0.081
BRI	Q1 (<2.945)	1 (Ref)		1 (Ref)		1 (Ref)	
	Q2 (2.945–3.615)	1.293 (0.869, 1.933)	0.207	1.214 (0.817, 1.815)	0.339	1.140 (0.744, 1.753)	0.548
	Q3 (3.615–4.482)	1.845 (1.269, 2.709)	0.002	1.670 (1.147, 2.454)	0.008	1.295 (0.861, 1.962)	0.217
	Q4 (>4.482)	2.468 (1.718, 3.593)	<0.001	2.109 (1.457, 3.089)	<0.001	1.449 (0.959, 2.207)	0.081
CI	Q1 (<1.222)	1 (Ref)		1 (Ref)		1 (Ref)	
	Q2 (1.222–1.271)	1.314 (0.906, 1.915)	0.152	1.292 (0.890, 1.885)	0.180	1.110 (0.741, 1.669)	0.614
	Q3 (1.271–1.321)	1.485 (1.032, 2.151)	0.034	1.413 (0.981, 2.049)	0.065	1.180 (0.793, 1.765)	0.416
	Q4 (>1.321)	1.783 (1.247, 2.572)	0.002	1.626 (1.132, 2.353)	0.009	1.280 (0.858, 1.919)	0.229
LAP	Q1 (<11.211)	1 (Ref)		1 (Ref)		1 (Ref)	
	Q2 (11.211–20.227)	1.828 (1.206, 2.807)	0.005	1.715 (1.134, 2.627)	0.012	1.65 (1.059, 2.599)	0.028
	Q3 (20.227–37.687)	2.715 (1.829, 4.105)	<0.001	2.462 (1.659, 3.718)	<0.001	1.957 (1.274, 3.051)	0.003
	Q4 (>37.687)	3.337 (2.259, 5.029)	<0.001	2.919 (1.970, 4.407)	<0.001	1.821 (1.180, 2.852)	0.008
VAI	Q1 (<0.690)	1 (Ref)		1 (Ref)		1 (Ref)	
	Q2 (0.690–1.132)	1.352 (0.912, 2.015)	0.135	1.398 (0.945, 2.081)	0.095	1.254 (0.821, 1.925)	0.297
	Q3 (1.132–1.949)	1.949 (1.349, 2.849)	<0.001	1.601 (1.091, 2.369)	0.017	1.286 (0.847, 1.965)	0.240
	Q4 (>1.949)	2.354 (1.645, 3.414)	<0.001	2.575 (1.795, 3.743)	<0.001	1.735 (1.162, 2.617)	0.008
CVAI	Q1 (<61.339)	1 (Ref)		1 (Ref)		1 (Ref)	
	Q2 (61.339–88.559)	1.396 (0.939, 2.089)	0.101	1.345 (0.906, 2.009)	0.144	1.425 (0.933, 2.191)	0.103
	Q3 (88.559–122.368)	2.013 (1.384, 2.962)	<0.001	1.827 (1.255, 2.689)	0.002	1.637 (1.091, 2.481)	0.019
	Q4 (>122.368)	2.466 (1.705, 3.612)	<0.001	2.137 (1.471, 3.143)	<0.001	1.547 (1.023, 2.36)	0.040

Abbreviations are the same as in **Table 1**. HR, hazard ratio; CIs, confidence intervals.

Model 1 was unadjusted. Model 2 was adjusted for age, educational levels, places of residence, drink history, smoke history, marital status, and SBP. Model 3 was further adjusted for history of hypertension, LDL, CRP, creatinine, BUN, FPG, TG, and SUA for BMI, WC, BRI, WHtR, and CI and for LAP, VAI, and CVAI, without TG.

The associations between adiposity indices with incident HUA in women are shown in **Table 4**. After adjusting for potential covariates, all adiposity indices were significantly associated with incident HUA. Compared with the first quartile, the HRs for HUA in the highest quartile of the adiposity indices were 3.550 (95%CIs, 2.173 to 5.989) for CVAI, 2.099 (95%CIs, 1.396 to 3.204) for VAI, 3.616 (95%CIs, 2.258 to 5.996) for LAP, 3.370 (95%CIs, 2.136 to 5.485) for BMI, 2.596 (95%CIs, 1.668 to 4.136) for WC, 3.206 (95%CIs, 1.976 to 5.391) for WHtR, 3.206 (95%CIs, 1.976 to 5.391) for BRI, and 1.673 (95%CIs, 1.089 to 2.602) for CI. The sensitivity analyses showed a similar association with the primary analyses in that all adiposity indices except CI were significantly associated with incident HUA (**Supplementary Table 1**).

**Table 4:** Association between adiposity indices with incident hyperuricemia multivariate logistic regression analysis in female participants.

		Model 1		Model 2		Model 3	
		HR (95%CIs)	*p*-Value	HR (95%CIs)	*p*-Value	HR (95%CIs)	*p*-Value
BMI	Q1 (<21.436)	1 (Ref)		1 (Ref)		1 (Ref)	
	Q2 (21.436–23.766)	2.390 (1.542, 3.793)	<0.001	2.664 (1.707, 4.254)	<0.001	2.196 (1.368, 3.604)	0.001
	Q3 (23.766–26.440)	2.549 (1.652, 4.032)	<0.001	2.846 (1.827, 4.541)	<0.001	1.948 (1.211, 3.203)	0.007
	Q4 (>26.440)	4.554 (3.036, 7.046)	<0.001	5.162 (3.387, 8.107)	<0.001	3.089 (1.949, 5.026)	<0.001
WC	Q1 (<78.50)	1 (Ref)		1 (Ref)		1 (Ref)	
	Q2 (78.50–86.00)	1.788 (1.150, 2.830)	0.011	1.807 (1.160, 2.867)	0.010	1.539 (9.580, 2.513)	0.079
	Q3 (86.00–92.60)	2.571 (1.679, 4.022)	<0.001	2.471 (1.608, 3.881)	<0.001	1.821 (1.145, 2.951)	0.013
	Q4 (>92.60)	4.581 (3.090, 6.991)	<0.001	4.232 (2.834, 6.502)	<0.001	2.596 (1.668, 4.136)	<0.001
WHtR	Q1 (<0.515)	1 (Ref)		1 (Ref)		1 (Ref)	
	Q2 (0.515–0.560)	2.348 (1.441, 3.944)	<0.001	2.333 (1.430, 3.924)	<0.001	2.015 (1.201, 3.475)	0.009
	Q3 (0.560–0.606)	4.228 (2.691, 6.913)	<0.001	3.994 (2.534, 6.547)	<0.001	3.091 (1.902, 5.205)	<0.001
	Q4 (>0.606)	5.844 (3.767, 9.462)	<0.001	5.207 (3.327, 8.494)	<0.001	3.206 (1.976, 5.391)	<0.001
BRI	Q1 (<3.636)	1 (Ref)		1 (Ref)		1 (Ref)	
	Q2 (3.636–4.544)	2.348 (1.441, 3.944)	<0.001	2.333 (1.430, 3.924)	<0.001	2.015 (1.201, 3.475)	0.009
	Q3 (4.544–5.565)	4.228 (2.691, 6.913)	<0.001	3.994 (2.534, 6.547)	<0.001	3.091 (1.902, 5.205)	<0.001
	Q4 (>5.565)	5.844 (3.767, 9.462)	<0.001	5.207 (3.327, 8.494)	<0.001	3.206 (1.976, 5.391)	<0.001
CI	Q1 (<1.240)	1 (Ref)		1 (Ref)		1 (Ref)	
	Q2 (1.240–1.300)	1.375 (0.905, 2.107)	0.138	1.356 (0.890, 2.083)	0.159	1.137 (0.724, 1.797)	0.578
	Q3 (1.300–1.357)	2.170 (1.477, 3.238)	<0.001	2.027 (1.372, 3.040)	<0.001	1.548 (1.015, 2.392)	0.045
	Q4 (>1.357)	2.902 (2.005, 4.278)	<0.001	2.420 (1.636, 3.642)	<0.001	1.673 (1.089, 2.602)	0.020
LAP	Q1 (<20.866)	1 (Ref)		1 (Ref)		1 (Ref)	
	Q2 (20.866–33.892)	1.649 (1.008, 2.749)	0.049	1.592 (0.971, 2.658)	0.069	1.42 (0.845, 2.427)	0.191
	Q3 (33.892–56.491)	3.034 (1.945, 4.881)	<0.001	2.884 (1.842, 4.654)	<0.001	2.150 (1.336, 3.556)	0.002
	Q4 (>56.491)	6.313 (4.173, 9.920)	<0.001	5.918 (3.881, 9.363)	<0.001	3.37 (2.136, 5.485)	<0.001
VAI	Q1 (<1.168)	1 (Ref)		1 (Ref)		1 (Ref)	
	Q2 (1.168–1.895)	1.369 (0.928, 2.031)	0.115	0.829 (0.515, 1.326)	0.435	0.66 (0.399, 1.085)	0.102
	Q3 (1.895–3.133)	1.585 (1.086, 2.332)	0.018	2.009 (1.359, 3.013)	<0.001	1.560 (1.021, 2.415)	0.042
	Q4 (>3.133)	2.570 (1.809, 3.702)	<0.001	3.424 (2.371, 5.042)	<0.001	2.099 (1.396, 3.204)	<0.001
CVAI	Q1 (<74.526)	1 (Ref)		1 (Ref)		1 (Ref)	
	Q2 (74.526–96.724)	1.702 (1.024, 2.888)	0.043	1.653 (0.991, 2.812)	0.058	1.420 (0.832, 2.469)	0.204
	Q3 (96.724–122.642)	3.246 (2.055, 5.307)	<0.001	3.081 (1.935, 5.074)	<0.001	2.115 (1.294, 3.561)	0.004
	Q4 (>122.642)	7.156 (4.678, 11.419)	<0.001	6.587 (4.191, 10.758)	<0.001	3.550 (2.173, 5.989)	<0.001

Abbreviations are the same as in **Table 1**. HR, hazard ratio; CIs, confidence intervals.

Model 1 was unadjusted. Model 2 was adjusted for age, educational levels, places of residence, drink history, smoke history, marital status, and SBP. Model 3 was further adjusted for history of hypertension, LDL, CRP, creatinine, BUN, FPG, TG, and SUA for BMI, WC, BRI, WHtR, and CI and for LAP, VAI, and CVAI, without TG.

### Predictive ability of the adiposity indices to identify incident HUA stratified by gender

**Figure 2:**
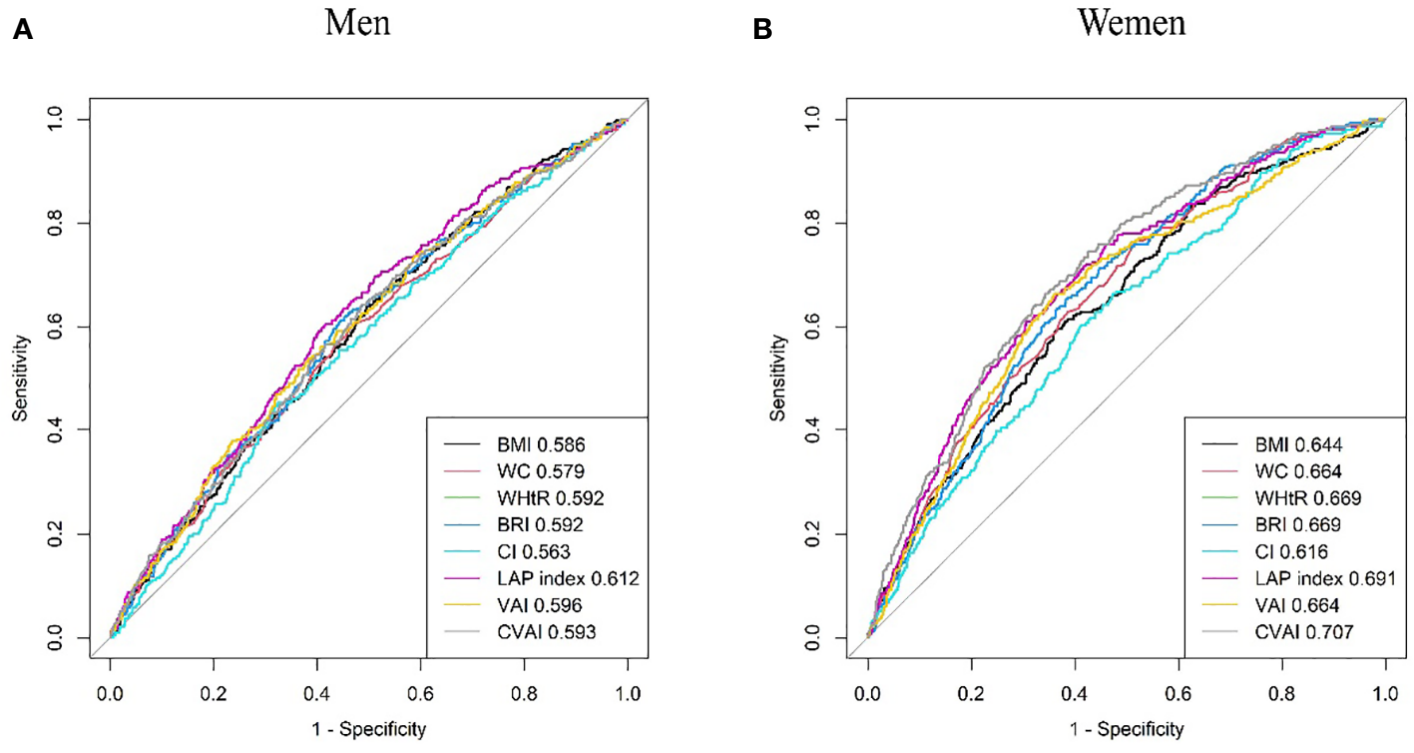
Comparison of the predictive value of eight adiposity indices for diagnosis of incident HUA in **(A)** men and **(B)** women. HUA, hyperuricemia.

Results from the ROC analysis and AUCs for the eight indices are shown in **Figure 2**. Results of the ROC analysis of the adiposity indices to identify incident HUA in male and female participants are shown in **Tables 5**, **6**, respectively. In male participants, LAP had the highest AUC (0.612), followed by CVAI (0.596), VAI (0.593), BRI (0.592), WHtR (0.592), BMI (0.586), WC (0.579), and CI (0.563). In female participants, CVAI had the highest AUC (0.707), followed by LAP (0.691), BRI (0.669), WHtR (0.669), VAI (0.664), WC (0.664), BMI (0.644), and CI (0.616) (all *p* < 0.001). The sensitivity analyses showed similar AUCs to the primary analyses (**Supplementary Table 2**).

**Table 5:** Area under the curve (AUC), cutoff value, sensitivity, specificity, and Youden index of eight adiposity indices in male participants.

Adiposity indices	AUC (95%CIs)	*p*-Value	Cutoff value	Sensitivity	Specificity	Youden index
BMI	0.586 (0.552, 0.619)	<0.001	22.443	0.639	0.504	0.143
WC	0.579 (0.545, 0.614)	<0.001	84.050	0.605	0.530	0.135
WHtR	0.592 (0.558, 0.626)	<0.001	0.519	0.611	0.555	0.166
BRI	0.592 (0.558, 0.626)	<0.001	3.728	0.611	0.555	0.166
CI	0.563 (0.529, 0.597)	<0.001	1.303	0.453	0.676	0.129
LAP	0.612 (0.579, 0.645)	<0.001	23.553	0.605	0.584	0.189
VAI	0.596 (0.562, 0.630)	<0.001	1.372	0.537	0.617	0.154
CVAI	0.593 (0.559, 0.627)	<0.001	88.454	0.642	0.515	0.157

Abbreviations are the same as in **Table 1**. CIs, confidence intervals.

**Table 6:** Area under the curve (AUC), cutoff value, sensitivity, specificity, and Youden index of eight adiposity indices in female participants.

Adiposity indices	AUC (95%CIs)	*p*-Value	Cutoff value	Sensitivity	Specificity	Youden index
BMI	0.644 (0.611, 0.678)	<0.001	24.881	0.592	0.638	0.230
WC	0.664 (0.632, 0.696)	<0.001	84.850	0.766	0.483	0.249
WHtR	0.669 (0.638, 0.700)	<0.001	0.576	0.652	0.617	0.269
BRI	0.669 (0.638, 0.700)	<0.001	4.890	0.652	0.617	0.269
CI	0.616 (0.583, 0.650)	<0.001	1.316	0.603	0.588	0.191
LAP	0.691 (0.660, 0.723)	<0.001	46.747	0.610	0.694	0.304
VAI	0.664 (0.630, 0.697)	<0.001	2.331	0.663	0.639	0.302
CVAI	0.707 (0.676, 0.737)	<0.001	109.471	0.663	0.657	0.320

Abbreviations are the same as in **Table 1**. CIs, confidence intervals.

### Incidence of HUA according to adiposity indices

**Figure 3:**
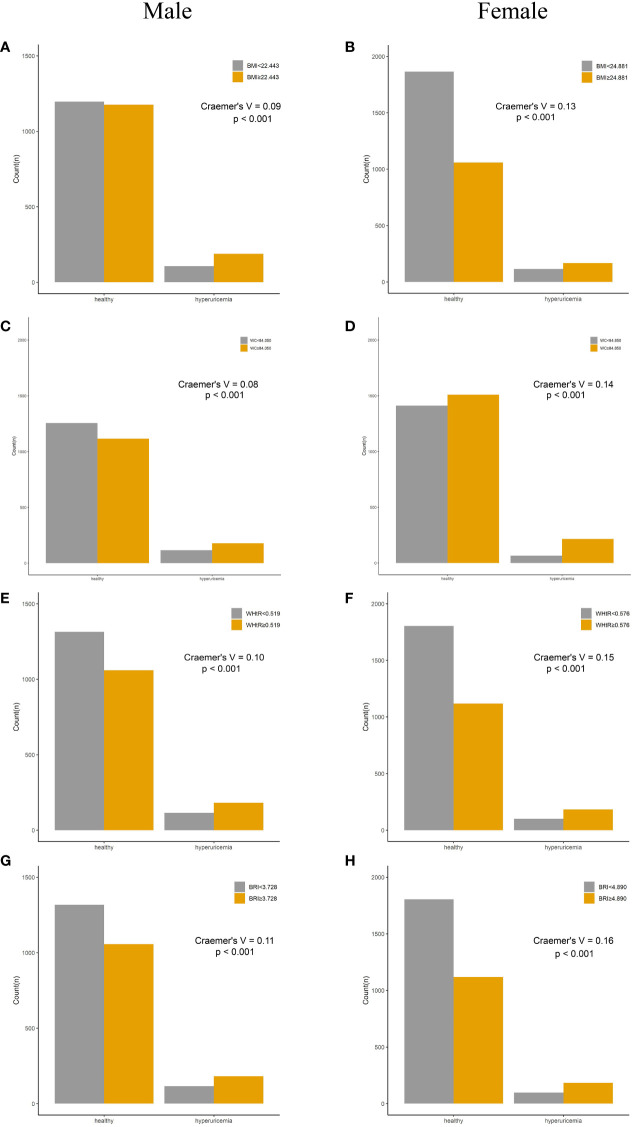
Incidence of HUA according to adiposity index cutoff values in men and women. **(A, B)** BMI. **(C, D)** WC. **(E, F)** WHtR. **(G, H)** BRI. HUA, hyperuricemia; BMI, body mass index; WC, waist circumference; WHtR, waist-to-height ratio; BRI, body roundness index.

**Figure 4:**
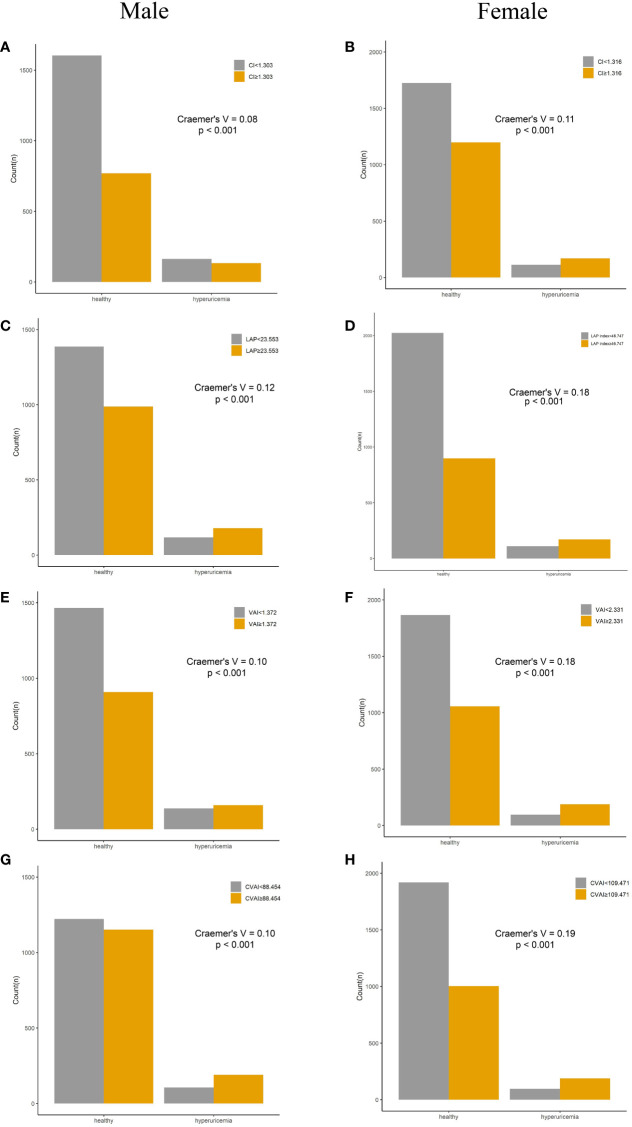
Incidence of HUA according to adiposity index cutoff values in men and women. **(A, B)** CI. **(C, D)** LAP. **(E, F)** VAI. **(G, H)** CVAI. HUA, hyperuricemia; CI, conicity index; LAP, lipid accumulation product; VAI, visceral adiposity index; CVAI, Chinese visceral adiposity index.

Compared with their counterparts, participants with high levels of adiposity indices had a significantly higher risk of HUA in both men and women. All eight adiposity indices had higher Cramer’s V to identify HUA in women than in men (**Figures 3**, **4**).

## Discussion

In this nationally prospective study, we investigated the relationships of adiposity indices with HUA risk in 5,873 Chinese middle-aged and older adults during 4 years of follow-up. The present study demonstrated that high visceral adiposity levels were positively associated with a higher risk of HUA among Chinese adults. All adiposity indices showed significant association with incident HUA in female participants, while only LAP, VAI, and CVAI, which contained lipid parameters, were significantly associated with incident HUA in male participants. Furthermore, all the adiposity indices showed better predictive power for incident HUA in women than men. CVAI had the highest AUC for the prediction of incident HUA, followed by LAP and VAI in the female participants, while LAP had the highest AUC for the prediction of incident HUA, followed by VAI in male participants. The results suggested that adiposity indices containing lipid parameters had stronger predictive power for incident HUA than other indices in both genders. In this study, the cumulative incidence of HUA was 9.8% in total, with 11.1% in men and 8.8% in women. Different HUA incidences have been reported in previous studies. For example, Zhang et al. showed that the cumulative incidence of hyperuricemia was 20.2% in total participants, with 21.3% in men and 16.6% in women, in an 8-year follow-up study (20), and 20.3% in total participants, with 27.7% in men and 13.2% in women in a prospective study performed in Tianjin, China, from 2013 to 2019 (26). The main reason for the lower incidence of HUA in our study compared with other studies was the shorter follow-up time. Consistent with previous studies, our study also demonstrated that men had a greater risk of developing HUA than women. This observation may be partially attributed to the fact that men typically exhibit higher baseline levels of uric acid compared to women (27). Moreover, men were more likely to drink alcohol, which had been significantly associated with HUA (28). Thus, more interventions should be taken to change the risk factors for HUA, especially in men.

The present study showed that several adiposity indices containing lipid parameters, such as CVAI, VAI, and LAP, were positively associated with incident HUA in both genders. Our results were consistent with several previous studies (11, 22, 23). Liu et al. reported that LAP was positively associated with HUA in China’s Yangtze River Delta region population (11), and Kahaer et al. found that LAP and VAI were positively associated with HUA in the Chinese Xinjiang population (16). Huang et al. reported that evaluated VAI increased the risk of hyperuricemia, independently of BMI and waist circumference, among middle-aged and elderly Chinese adults (21). Previous studies demonstrated that adiposity indices containing lipid parameters showed a better ability to identify obesity and the distribution of adipose tissue, especially visceral adiposity tissue (VAT), than those that did not contain lipid parameters (11, 14, 22). It was indicated that the distribution of adipose tissue instead of the amount had major effects on metabolism (29). Previous studies suggested that uric acid and metabolic syndrome were closely related and that such an association might be bidirectional (8). Some possible mechanisms might explain the effect of metabolic disorders on HUA. First, increased VAT accumulation caused the free flow of free fatty acids to the liver and the overproduction of very-low-density lipoprotein (VLDL) and TG. The increase in lipid synthesis increased the need for NADPH and then accelerated the pentose phosphate pathway, which led to the *de novo* purine synthesis, thus increasing the production of UA (20, 30). Second, the elevation of TG and/or TC lipid metabolism disorders may impair kidney function and result in decreased renal blood flow and reduction of the excretion and reabsorption of uric acid (31, 32). Third, previous studies demonstrated that VAT-induced adiponectin and leptin were significantly associated with insulin resistance, which could affect kidney functions, increase reabsorption of uric acid, decrease excretion of uric acid, and lead to hyperuricemia (33, 34).

Our results showed that several adiposity indices, including BMI, WC, WHtR, BRI, and CI, displayed gender-specific associations with incident HUA between men and women. The significant associations between these incidences and incident HUA were observed exclusively in women. Furthermore, all adiposity indices in the present study showed a higher predictive ability of HUA in women than in men. Previous studies reported inconsistent results on the association between these indices and HUA. Kahaer et al. reported that BMI, WC, WHtR, and BRI exhibited no significant association with HUA in both men and women (16), while Zhang et al. reported that BRI, WHtR, BMI, and WC were associated with HUA in both genders (15). Several previous studies also demonstrated that the adiposity indices had a greater predictive ability of HUA in women than men in longitudinal and cross-sectional studies (15, 16). The gender variation regarding the association between adiposity indices with HUA and the predictive ability may be explained by biological differences between men and women. Middle-aged and older women showed age-related increases in serum uric acid levels, while such a trend was not found in men (35). In the present study, the female participants were generally in perimenopause and menopause. Their estrogen levels decreased with aging (36, 37), thus reducing the renal clearance of urate and resulting in increased SUA levels (36). Furthermore, a clinical study reported that sex hormone replacement therapy can reduce SUA concentrations and decrease the risk of gout flare in postmenopausal women. This suggested that the decreased estrogen levels in postmenopausal women could increase the risk of HUA (38). Taken together, these physiological changes may cause women to be more sensitive to metabolic changes and thus more likely to develop HUA. In addition, the associations were explored in 4-year folloe-up study.

The present study has several strengths. The data in this study came from a population-based nationwide prospective survey, which provides a representative sample of the population. Then, a longitudinal study was conducted, whose evidence power was stronger than that of previous cross-sectional studies. Furthermore, the predictive ability of a total of eight adiposity indices with HUA was investigated, and for the first time, the associations between several adiposity indices (such as CVAI, VAI, and LAP) and incident HUA were reported in a longitudinal survey. In addition, we also proposed optimal cutoff values for these indices by ROC curve analysis and further verified the effectiveness in the diagnosis of HUA. Nevertheless, there are several limitations in this study. First, although we have adjusted for several potential confounding variables, we did not include data on variables that could potentially influence uric acid levels, such as dietary factors (consumption of dairy, meat, and micronutrients). Second, the use of uric acid-lowering drugs, such as allopurinol, was not available in the study, which may cause diagnostic bias. Third, the participants were Chinese adults aged ≥45 years, which may limit the generalizability of our results to the younger population and other ethnic groups.

In conclusion, our study provided strong evidence that the adiposity indices were effective predictors of incident HUA in general middle-aged and older Chinese adults. Furthermore, some indices, including BMI, WC, WHtR, BRI, and CI, displayed gender-specific associations with incident HUA, with a significant association observed exclusively in women. All adiposity indices showed better predictive ability for incident HUA in women than men. For the preventive prospect and clinical practice, adiposity indices, especially those containing lipid parameters, were obtainable and cost-effective and could be used in HUA prevention among Chinese middle-aged and older adults.

## Data availability statement

The datasets presented in this study can be found in online repositories. The names of the repository/repositories and accession number(s) can be found below: http://charls.pku.edu.cn/.

## Author contributions

ZL: Conceptualization, Writing – original draft. QZ: Conceptualization, Methodology, Writing – original draft. YT: Writing – original draft. JL: Writing – original draft. QC: Funding acquisition, Methodology, Writing – original draft. HY: Conceptualization, Formal analysis, Methodology, Software, Writing – original draft. SZ: Formal analysis, Funding acquisition, Writing – original draft.
