# Supplementary material for: Sex-specific differences in the associations between adiposity indices and incident hyperuricemia among middle-aged and older adults: a nationwide longitudinal study
Source: Front Endocrinol (Lausanne). 2024 Feb 9;15:1336471. doi: 10.3389/fendo.2024.1336471 (PMC10884268; doi:10.3389/fendo.2024.1336471)
Supplement: Supplementary file 1 [file Table_1.docx]

**Supplementary materials**

Table S1 Association of adiposity indices with incident hyperuricemia multivariate logistic regression analysis in female participants.

|  | | HR (95%CIs) | *P*-value |
| --- | --- | --- | --- |
| WHtR | Q1 | 1 (Ref) |  |
|  | Q2 | 1.634(0.771,3.594) | 0.207 |
|  | Q3 | 1.988(0.977,4.267) | 0.066 |
|  | Q4 | 2.711(1.385,5.663) | 0.005 |
| WC | Q1 | 1 (Ref) |  |
|  | Q2 | 1.64(0.782,3.581) | 0.199 |
|  | Q3 | 2.189(1.08,4.681) | 0.035 |
|  | Q4 | 2.775(1.422,5.779) | 0.004 |
| BMI | Q1 | 1 (Ref) |  |
|  | Q2 | 2.663(1.313,5.711) | 0.009 |
|  | Q3 | 1.875(0.904,4.084) | 0.100 |
|  | Q4 | 3.186(1.589,6.812) | 0.002 |
| CI | Q1 | 1 (Ref) |  |
|  | Q2 | 0.801(0.394,1.624) | 0.537 |
|  | Q3 | 1.306(0.695,2.512) | 0.413 |
|  | Q4 | 1.496(0.811,2.852) | 0.207 |
| BRI | Q1 |  |  |
|  | Q2 | 1.634(0.771,3.594) | 0.207 |
|  | Q3 | 1.988(0.977,4.267) | 0.066 |
|  | Q4 | 2.711(1.385,5.663) | 0.005 |
| LAP | Q1 | 1 (Ref) |  |
|  | Q2 | 1.26(0.563,2.899) | 0.577 |
|  | Q3 | 2.158(1.067,4.655) | 0.039 |
|  | Q4 | 2.237(1.138,4.725) | 0.026 |
| CVAI | Q1 | 1 (Ref) |  |
|  | Q2 | 3.028(1.228,8.468) | 0.023 |
|  | Q3 | 3.287(1.364,9.077) | 0.013 |
|  | Q4 | 4.87(2.069,13.249) | <0.001 |
| VAI | Q1 | 1 (Ref) |  |
|  | Q2 | 1.632(0.737,3.826) | 0.239 |
|  | Q3 | 2.811(1.349,6.358) | 0.008 |
|  | Q4 | 2.796(1.39,6.159) | 0.006 |

Abbreviations: BMI, body mass index; WC, waist circumference; WHtR, waist-to-height ratio; BRI, body roundness index; CI, conicity index; LAP, lipid accumulation product index; VAI, visceral adiposity index; CVAI, Chinese visceral adiposity index. HR, hazard ratio; CIs, confidence intervals.

Model was adjusted for age, educational levels, places of residence, drink history, smoke history, marital status, SBP, history of hypertension, LDL, CRP, creatine, BUN, FPG, TG, and SUA for BMI, WC, BRI, WHtR, and CI, and for LAP, VAI, and CVAI, without TG.

**Table S2.** Area under curve (AUC), cutoff value, sensitivity, specificity, and Youden index of eight adiposity indices in female participants.

| Adiposity indices | AUC (95%CIs) | *P*-value | Cut-off value | Sensitivity | Specificity | Youden index |
| --- | --- | --- | --- | --- | --- | --- |
| BMI | 0.620 (0.569,0.672) | <0.001 | 22.559 | 0.829 | 0.369 | 0.198 |
| WC | 0.655 (0.605,0.704) | <0.001 | 90.15 | 0.593 | 0.689 | 0.282 |
| WHtR | 0.647 (0.597,0.697) | <0.001 | 0.58 | 0.667 | 0.626 | 0.293 |
| BRI | 0.647 (0.597,0.697) | <0.001 | 4.988 | 0.667 | 0.626 | 0.293 |
| CI | 0.624 (0.573,0.676) | <0.001 | 1.316 | 0.626 | 0.58 | 0.206 |
| LAP | 0.681(0.634,0.727) | <0.001 | 33.334 | 0.805 | 0.499 | 0.304 |
| VAI | 0.676 (0.631,0.722) | <0.001 | 2.488 | 0.634 | 0.651 | 0.285 |
| CVAI | 0.704 (0.661,0.748) | <0.001 | 119.092 | 0.585 | 0.727 | 0.312 |

Abbreviations are the same as in Table S1.

|  | SUA | creatinine | CRP | BUN | TG | HDL | TC | LDL | SBP | DBP | HbA1c | FPG |
| --- | --- | --- | --- | --- | --- | --- | --- | --- | --- | --- | --- | --- |
| SUA | 1.00 | 0.37 | 0.04 | 0.14 | 0.18 | -0.11 | 0.12 | 0.06 | 0.12 | 0.09 | -0.02 | -0.02 |
| creatinine | 0.37 | 1.00 | 0.02 | 0.24 | 0.04 | 0.00 | 0.10 | 0.07 | 0.06 | 0.04 | -0.02 | 0.01 |
| CRP | 0.04 | 0.02 | 1.00 | -0.05 | 0.00 | -0.08 | -0.02 | 0.00 | 0.05 | 0.01 | 0.09 | 0.11 |
| BUN | 0.14 | 0.24 | -0.05 | 1.00 | 0.00 | 0.11 | 0.11 | 0.06 | 0.01 | 0.00 | 0.03 | 0.01 |
| TG | 0.18 | 0.04 | 0.00 | 0.00 | 1.00 | -0.53 | 0.23 | -0.16 | 0.13 | 0.12 | 0.16 | 0.28 |
| HDL | -0.11 | 0.00 | -0.08 | 0.11 | -0.53 | 1.00 | 0.20 | 0.16 | -0.10 | -0.12 | -0.09 | -0.17 |
| TC | 0.12 | 0.10 | -0.02 | 0.11 | 0.23 | 0.20 | 1.00 | 0.84 | 0.08 | 0.06 | 0.16 | 0.13 |
| LDL | 0.06 | 0.07 | 0.00 | 0.06 | -0.16 | 0.16 | 0.84 | 1.00 | 0.04 | 0.03 | 0.11 | 0.04 |
| SBP | 0.12 | 0.06 | 0.05 | 0.01 | 0.13 | -0.10 | 0.08 | 0.04 | 1.00 | 0.70 | 0.07 | 0.10 |
| DBP | 0.09 | 0.04 | 0.01 | 0.00 | 0.12 | -0.12 | 0.06 | 0.03 | 0.70 | 1.00 | 0.05 | 0.07 |
| HbA1c | -0.02 | -0.02 | 0.09 | 0.03 | 0.16 | -0.09 | 0.16 | 0.11 | 0.07 | 0.05 | 1.00 | 0.72 |
| FPG | -0.02 | 0.01 | 0.11 | 0.01 | 0.28 | -0.17 | 0.13 | 0.04 | 0.10 | 0.07 | 0.72 | 1.00 |

Table S3 Pearson's correlation coefficient between variables in male participants

Abbreviations: SBP, systolic blood pressure; DBP, diastolic blood pressure; TG, triglycerides; HDL, high-density lipoprotein; TC, total cholesterol; LDL, low-density lipoprotein; FPG, fasting plasma glucose; HbA1c, glycosylated hemoglobin; CRP, C-reactive protein; BUN, blood urea nitrogen; SUA, serum uric acid;

Table S4 Pearson's correlation coefficient between variables in female participants

|  | SUA | creatinine | CRP | BUN | TG | HDL | TC | LDL | SBP | DBP | HbA1c | FPG |
| --- | --- | --- | --- | --- | --- | --- | --- | --- | --- | --- | --- | --- |
| SUA | 1.00 | 0.37 | 0.03 | 0.12 | 0.15 | -0.02 | 0.16 | 0.08 | 0.11 | 0.08 | -0.05 | -0.05 |
| creatinine | 0.37 | 1.00 | 0.00 | 0.20 | 0.05 | -0.07 | 0.09 | 0.09 | 0.09 | 0.04 | 0.04 | 0.02 |
| CRP | 0.03 | 0.00 | 1.00 | 0.00 | -0.01 | -0.04 | -0.02 | -0.01 | -0.01 | -0.02 | 0.03 | 0.03 |
| BUN | 0.12 | 0.20 | 0.00 | 1.00 | -0.04 | 0.11 | 0.06 | 0.04 | 0.01 | -0.03 | 0.00 | 0.01 |
| TG | 0.15 | 0.05 | -0.01 | -0.04 | 1.00 | -0.42 | 0.23 | -0.16 | 0.06 | 0.12 | 0.16 | 0.27 |
| HDL | -0.02 | -0.07 | -0.04 | 0.11 | -0.42 | 1.00 | 0.23 | 0.05 | -0.03 | -0.06 | -0.09 | -0.10 |
| TC | 0.16 | 0.09 | -0.02 | 0.06 | 0.23 | 0.23 | 1.00 | 0.81 | 0.09 | 0.11 | 0.10 | 0.08 |
| LDL | 0.08 | 0.09 | -0.01 | 0.04 | -0.16 | 0.05 | 0.81 | 1.00 | 0.08 | 0.06 | 0.06 | -0.03 |
| SBP | 0.11 | 0.09 | -0.01 | 0.01 | 0.06 | -0.03 | 0.09 | 0.08 | 1.00 | 0.75 | 0.08 | 0.10 |
| DBP | 0.08 | 0.04 | -0.02 | -0.03 | 0.12 | -0.06 | 0.11 | 0.06 | 0.75 | 1.00 | 0.07 | 0.06 |
| HbA1c | -0.05 | 0.04 | 0.03 | 0.00 | 0.16 | -0.09 | 0.10 | 0.06 | 0.08 | 0.07 | 1.00 | 0.63 |
| FPG | -0.05 | 0.02 | 0.03 | 0.01 | 0.27 | -0.10 | 0.08 | -0.03 | 0.10 | 0.06 | 0.63 | 1.00 |

Abbreviations: SBP, systolic blood pressure; DBP, diastolic blood pressure; TG, triglycerides; HDL, high-density lipoprotein; TC, total cholesterol; LDL, low-density lipoprotein; FPG, fasting plasma glucose; HbA1c, glycosylated hemoglobin; CRP, C-reactive protein; BUN, blood urea nitrogen; SUA, serum uric acid;
